# Punicalagin Protects Diabetic Nephropathy by Inhibiting Pyroptosis Based on TXNIP/NLRP3 Pathway

**DOI:** 10.3390/nu12051516

**Published:** 2020-05-22

**Authors:** Xin An, Yahui Zhang, Yuan Cao, Jihua Chen, Hong Qin, Lina Yang

**Affiliations:** Xiangya School of Public Health, Central South University, Changsha 410128, China; 2501130307@csu.edu.cn (X.A.); yahuizhang@csu.edu.cn (Y.Z.); caoyuan2017@csu.edu.cn (Y.C.); chenjh@csu.edu.cn (J.C.); qinhong@csu.edu.cn (H.Q.)

**Keywords:** punicalagin, diabetic nephropathy, pyroptosis, TXNIP, NLRP3 inflammasome

## Abstract

Diabetic nephropathy is a diabetic complication caused by chronic inflammation. As the primary polyphenol in pomegranate, punicalagin is believed to have significant anti-inflammatory properties. In this study, we established a mice model for diabetes induced by high-fat diet (HFD)/ streptozotocin (STZ) to verify the protective effect of punicalagin in vivo. The results show that the blood urea nitrogen (BUN), serum creatinine (CREA), and the urine albumin to creatinine ratio (UACR) were significantly decreased in diabetic mice after punicalagin intervention, and the symptoms of glomerular interstitial hyperplasia and glomerular hypertrophy were alleviated. Pyroptosis is an essential manner of programmed cell death in the inflammatory response; the expression of pyroptosis-related proteins such as interleukin-1 (IL-1β), cysteinyl aspartate-specific protease-1 (caspase-1), gasdermin D (GSDMD), and nucleotide-binding oligomerization domain, leucine-rich repeat and pyrin domain containing protein 3 (NLRP3) was decreased in our study, which proved that the administration of punicalagin for eight weeks can significantly inhibit pyroptosis in mice. In addition, punicalagin reduced high glucose-mediated protein expressions of nicotinamide adenine dinucleotide phosphate (NADPH) oxidase 4 (NOX4) and alleviated mitochondria damage. Low expression of NOX4 inhibits the dissociation of thioredoxin (Trx) and thioredoxin-interacting protein (TXNIP) and the suppression of NLRP3 inflammasome activation. To summarize, our study provided evidence that punicalagin can alleviate diabetic nephropathy, and the effect is associated with downregulating the expression of NOX4, inhibiting TXNIP/NLRP3 pathway-mediated pyroptosis, suggesting its therapeutic implications for complications of diabetes.

## 1. Introduction

Diabetic nephropathy (DN) is one of the complications of diabetes, the main clinical manifestations of which are characterized by proteinuria, hypertension, and a gradual decrease in renal function [1]. Diabetic nephropathy appears in 30% to 50% of end-stage renal disease cases and has become one of the public problems that severely threaten the health of people worldwide [2].

Although DN has been mainly considered a non-immune renal disease in the past, recent theoretical and experimental studies have shown that chronic inflammation plays a vital part in the occurrence and development of DN [3]. The inflammatory mechanism is a key factor for its continued development based on metabolic disorders and hemodynamic mechanisms. A diabetic environment leads to the production and circulation of advanced glycation end products and changes in hemodynamics and hormones, followed by the release of reactive oxygen species (ROS) and inflammatory mediators [4]. 

Pyroptosis is a recently identified mode of programmed cell death accompanied by an inflammatory response [5]. This new type of pro-inflammatory programmed cell death is often mediated by caspase-1, which is also associated with the release of a large number of inflammatory factors, eventually inducing a cascade amplification of the inflammatory response [6]. The activation of intracellular caspase-1 by stimulation signals depends on inflammasomes including NLRP3 [7]. The NLRP3 receptor is a part of the nucleotide binding oligomerization domain (NOD)-like receptors (NLRs) family. It can recognize the activation of stimulus signals to form an NLRP3 inflammasome, which is caused by a series of cascade signals. As a powerful inflammatory mediator, IL-1β can induce the expression of other inflammatory factors, leading to cell death, and is closely associated with the occurrence of DN [8]. Related studies have shown that NLRP3 recognizes non-microbiological danger signals and mediates pyroptosis. It plays an essential role in the development of various non-bacterial inflammatory diseases, especially those closely related to metabolic disorders and endogenous stimuli [9]. In the state of hyperglycemia, the body can generate a variety of dangerous related molecules such as fatty acids, ROS, etc., which can be recognized by related pattern recognition receptors (PRR), thereby initiating pyroptosis [10]. Upon the activation of NLRP3 inflammasomes, NLRP3 activates caspase-1, and then IL-1β and interleukin-18 (IL-18) are processed and activated, resulting in pro-inflammatory responses. In general, inhibition of the NLRP3 inflammasome can slow the occurrence of pyroptosis in diabetes and related complications, which may be the key to preventing and slowing the development of DN.

The Trx system is an important antioxidant system, which resists oxidative stress by providing electrons to peroxiredoxins so that they may remove reactive oxygen and nitrogen species efficiently [11]. In addition, the Trx system can also play an important role in cell death and immune response through interaction with TXNIP [12]. Excessive production of mitochondrial ROS (mtROS) results in the dissociation of TXNIP from its binding protein Trx, which then binds to NLRP3 and causes the activation of the NLRP3 inflammasome. It has been reported that NADPH oxidase (NOX4)-derived ROS production experiments promote the dissociation of TXNIP-Trx and increase the combination of TXNIP with NLRP3 in vivo and in vitro [13]. TXNIP might be the key to linking the hyperglycemic environment to inflammation by activating the NLRP3 inflammasome, an important part of inflammation. TXNIP probably plays a significant role in this approach in DN as well [14].

Polyphenols are natural antioxidants and are found in a variety of human dietary components such as fruits, vegetables, and tea, and play a protective role in many chronic diseases, including diabetes, cardiovascular disease, and neurodegenerative diseases [15]. Numerous studies have identified broad biological properties that polyphenols possess, including but not limited to immune-modulatory, anti-inflammatory, and antioxidant actions. Existing studies have found that the biological effects of polyphenols may be mediated through their interactions with particular proteins [16]. The protective effect of polyphenols on diabetic nephropathy is not rare in existing studies. For example, bergenin, a plant polyphenol, can inhibit glucose-induced extracellular matrix (ECM) production in glomerular mesangial cells [17]; a phenolic aqueous extract called mangosteen vinegar rind could significantly modulate pathological alterations of diabetic mice’s kidneys [18].

Inflammation is believed to be the main cause of many human diseases such as cancer, type 2 diabetes, arthritis, and cardiovascular diseases [19]. Polyphenols that originate from plants have shown anti-inflammatory activity in vitro and in vivo, stressing their special advantage as therapeutic tools for a variety of acute and chronic diseases. Current research has shown that polyphenols can protect against diabetic kidney injury, which may be related to their anti-inflammatory effect. Other studies have shown that pomegranate peel extract has protective efficacy in reducing STZ-induced glomerular sclerosis and renal fibrosis [20].

Pomegranate is a widely cultivated and edible fruit that contains a variety of polyphenols. Punicalagin (PU) is the main component of pomegranate polyphenols [21]. It has been reported to increase superoxide dismutase (SOD) production and reduce ROS and nitric oxide (NO) production, suppressing the Lipopolysaccharide (LPS)-induced inflammatory response in macrophages [22]. Recently, our research team showed that PU can protect against the complications of diabetes [23].

In this study, we investigated the intervention effect of PU on diabetic nephropathy in a mice model induced by high-fat diet and STZ to prove the protective effect of PU on diabetic nephropathy and explore the possible mediating role taken by the TXNIP/NLRP3 pathway in the pyroptosis mechanism.

## 2. Materials and Methods

### 2.1. Reagents and Antibodies

Punicalagin (purity = 98%, CAS: 65995-63-3) was purchased from Herbpurify Co., Ltd. (Chengdu, China). A urea assay kit, a creatinine assay kit (sarcosine oxidase) and a urine microalbumin assay kit were purchased from the Nanjing Jiancheng Bioengineering Institute (China). A mitochondrial membrane potential assay kit with JC-1 and a tissue mitochondria isolation kit were purchased from Beyotime Biotechnology (Shanghai, China). Antibodies targeting the following proteins were used in this study: NLRP3 (A12694, Abclonal), Trx (A01219-1, BOSTER), TXNIP (sc-166234, Santa Cruz; A9342, Abclonal), IL-1β (A1112, Abclonal), caspase-1 (BM4291, BOSTER), GSDMD (A10164, Abclonal), β-actin (60008-1-Ig, Proteintech), NOX4 (14347-1-AP, Proteintech).

### 2.2. Animals and Treatment

Eight-week-old male C57BL/6J mice (from Central South University, Changsha, China) (SCXK2016-002) were housed at 55% ± 5% humidity and constant room temperature (24 °C), under a controlled light cycle. All animals were free to drink water. The animal procedures were in accordance with the NIH laboratory animal care and usage guidelines and the standards set by the Institutional Review Board of Xiangya School of Public Health, Central South University.

The mice model of DN was established by the administration of a high-fat diet (HFD) and the intraperitoneal injection of streptozotocin (STZ) solution (dissolved in a 0.01 M citrate buffer, pH 4.5; Solarbio Science and Technology Co. Ltd., Beijing, China) with the dose of 100 mg/kg body weight. Seventy-two hours after the injection, fasting blood glucose (FBG) was measured by a blood glucose meter (Bayer). The mice were identified as successfully prepared in cases where random blood glucose level ≥ 16.7 mmoL/L. Mice injected with equivalent amounts of buffer solution were used as controls (*n* = 8). The model mice were randomly divided into a DN group (*n* = 8) and a DN + PU group (*n* = 8). The mice in the DN + PU group were given PU utilizing intragastric administration once a day for 8 weeks (20mg/kg body weight/day), and the mice in the DN groups were injected with an equal volume of distilled water. After 8 weeks, all the mice were weighed and sacrificed; the mice were transferred to a metabolic cage 2 days before they were executed. Urine samples and blood were collected for subsequent tests. After the kidneys were removed and weighed, the kidney coefficient (kidney weight (g)/mouse weight (g)) was calculated. Then the kidneys were saved for periodic acid-Schiff (PAS) staining, Masson staining and periodic acid-silver methenamine (PASM) staining. This study was approved by the Institutional Animal Care and Use Committee of Xiangya School of Public Health of Central South University, and abided by the Guide for the Care and Use of Laboratory Animals by the National Institute of Health.

### 2.3. Biochemical Examination

The urease conductivity rate method was used for determining blood urea nitrogen (BUN). Serum creatinine (CREA) was measured using picric acid and the urine albumin to creatinine ratio (UACR) by radioimmunoassay.

### 2.4. Determination of Glomerular Tuft Area

PAS staining sections were taken for histomorphology observation, and image analysis software (ImageJ) was used for measurement. The glomerular tuft area was measured under a 400× visual field. Five glomeruli were measured from each section, and their average value was recorded.

### 2.5. Analysis of Mitochondrial Membrane Potential

JC-1 is a monomer that emits green light when excited by blue light. At high membrane potential, the JC-1 monomer in the cell is transformed into JC-1 aggregate in the mitochondrial matrix, and red light is emitted under the excitation of green light. In short, after using a tissue mitochondrial separation kit to extract the mitochondria of renal tissue, 0.1 mL of purified mitochondria with a total protein amount of 10–100 μg was added to 0.9 mL of 5 times diluted JC-1 working solution. After mixing, a fluorescence spectrophotometer was used for the time scan. The emission wavelength was 485 nm and the excitation wavelength was 590 nm. 

### 2.6. Western Blotting

Total proteins were extracted from kidney tissues. In this process, 10% or 15% sodium dodecyl sulfate-polyacrylamide gel electrophoresis (SDS-PAGE) gels were used to separate the denatured proteins (30 μg), which were then transferred to a polyvinylidene difluoride membrane. The membranes were blocked for 1 h and then incubated separately with different kinds of primary antibodies against β-actin, NLRP3, GSDMD, caspase-1, IL-1β, NOX4, Trx, and TXNIP at 4 °C overnight. After washing with a buffer and incubation with the corresponding secondary antibody, each membrane was treated with BeyoECL Star chemiluminescent reagent. The protein bands were detected by a chemiluminescence image analysis system (Tanon Science and Technology Co. Ltd., Shanghai, China), and their intensities were measured with ImageJ software. The expression levels of the target protein were analyzed using a semi-quantitative method. 

### 2.7. Statistical Analysis

SPSS 18.0 software (IBM Corp, Armonk, New York, USA) was used for statistical analysis. All data are expressed as mean ± SD. Statistical analysis was performed using unpaired Student’s t-tests to compare between two groups. One-way ANOVA was used for multi-group comparisons. A *p*-value of <0.05 was considered statistically significant.

## 3. Results

### 3.1. PU Improves Renal Function

**Figure 1:**
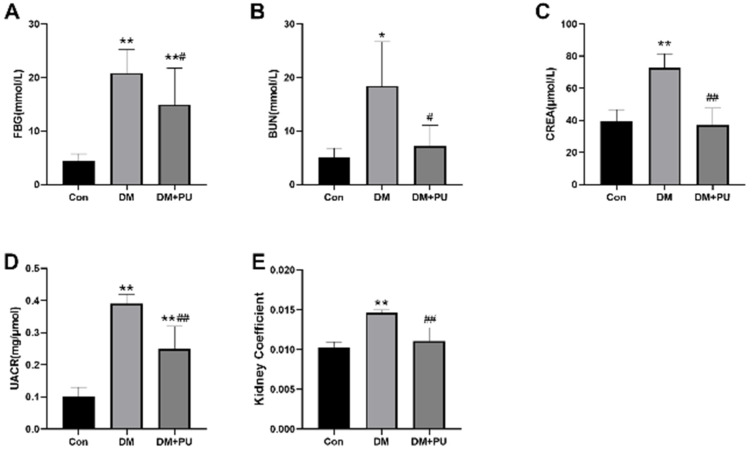
Effects of PU on the HFD/STZ-induced diabetic mice model after 8 weeks. (**A**) Fasting blood glucose intragastric administration. (**B**) Blood urea nitrogen after 8 weeks’ intragastric administration. (**C**) Serum creatinine after 8 weeks’ intragastric administration. (**D**) Urine albumin-creatine ratio after 8 weeks’ intragastric administration. (**E**) Kidney coefficient of the mice after 8 weeks’ intragastric administration. * *p* < 0.05 vs. control (Con) group; ** *p* < 0.01 vs. Con group; # *p* < 0.05 vs. diabetes mellitus (DM) group; ## *p* < 0.01 vs. DM group; *n* = 8.

To investigate the protective effects of PU on DN, we used an HFD/STZ model for in vivo experiments. HFD/STZ-induced diabetic mice showed significant renal dysfunction, which was confirmed by the increase in the ratio of urine albumin to creatinine and the levels of serum urea nitrogen and creatinine. The levels of blood urea BUN, CREA, and UACR in diabetic mice were significantly reduced after eight weeks of intervention with PU (Figure 1A–D). The results showed that the kidney coefficient of the model group was significantly higher than that of the control group. In contrast, the kidney coefficient of the PU-treated group was remarkably lower than that of the model group (Figure 1E). All in all, these data suggested that PU improves renal function in diabetic mice.

### 3.2. PU Alleviates Pathological Changes in the Kidney

**Figure 2:**
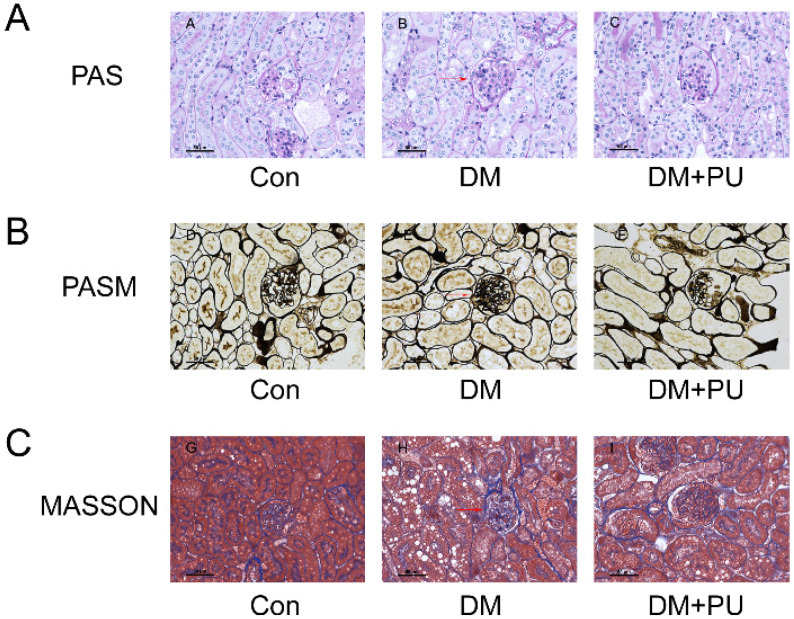
Changes in renal histology of kidney damage. (**A**) PAS sections of the kidney tissues after 8 weeks’ intragastric administration. (**B**) PASM sections of the kidney tissues after 8 weeks’ intragastric administration. (**C**) Masson sections of the kidney tissues after 8 weeks’ intragastric administration (magnification × 400).

In our experiment, PAS staining of the model group showed the typical pathological changes of diabetic nephropathy (DN), such as apparent mesangial matrix hyperplasia, renal vesicles, and glomerular basement membrane thickening. The intervention of PU can reduce such pathological changes (Figure 2A). Measurement of the glomerular tuft area showed that the glomerulus in the diabetes mellitus (DM) group was hypertrophic, while that in the PU-treatment group was closer to the normal size (Table 1). Additionally, Masson staining showed that glomerular collagen deposition and fibrosis in the model group were more serious compared with other groups. At the same time, treatment with PU significantly inhibited glomerular fibrosis (Figure 2C). The results of PASM staining of the kidneys show that the glomeruli of the model group were sclerotic and bulky, with mesangial matrix hyperplasia, renal vesicles, and glomerular basement membrane thickening compared with the control group (Figure 2B). In contrast, these abnormal changes were significantly relieved in the PU-treated group.

### 3.3. Effect of PU on Pyroptosis-Mediated Inflammation in Diabetic Mice

**Figure 3:**
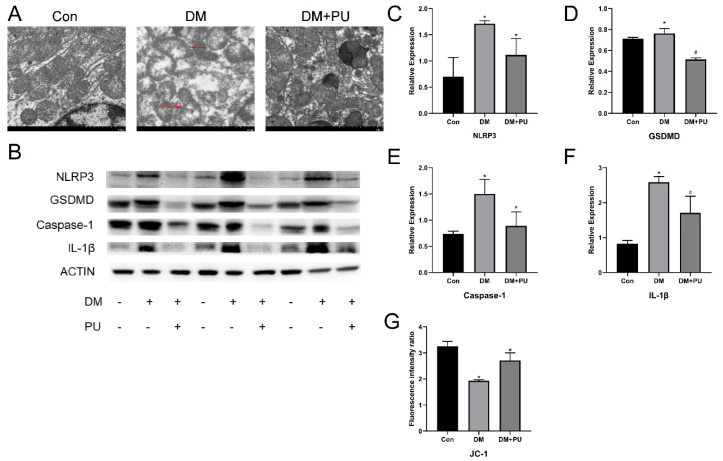
Effects of PU on mitochondrion injury and pyroptosis of the diabetic mice’s kidneys. (**A**) Representative image of the electron microscopy analysis, significant mitochondrial fragmentation and damage (magnification × 7000). (**B**) Protein expression levels of NLRP3 by western blotting. (**C**) Protein expression levels of GSDMD by western blotting. (**D**) The results of NLRP3, GSDMD, caspase-1, and IL-1β by western blotting. (**E**) Expression levels of caspase-1 by western blotting. (**F**) Expression levels of IL-1β by western blotting. (**G**) Level of mitochondrial membrane potential detected by JC-1 fluorophore. The relative expression is expressed as the ratio of the target protein to β-actin. * *p* < 0.05 vs. Con group; # *p* < 0.05 vs. DM group.

We further analyzed the anti-inflammatory effect of PU in diabetic nephropathy. As shown in Figure 3, compared with normal mice, the presence of the pyroptosis-related inflammatory factor IL-1 β in the kidney tissue of HFD/STZ-induced diabetic mice increased significantly, and the release of IL-1β in kidney tissue was inhibited after PU treatment (Figure 3F). In the model group, the abatement of mitochondrial membrane potential reveals the damage to mitochondria in the renal tissue, which was confirmed by TEM observation of renal tissue sections (Figure 3A,G). The renal tissue in the model group clearly showed glomerular basement membrane thickening and the characteristics of pyroptosis compared with the control group, such as swelling of the mitochondria, perforation and dissolution of the cell membrane, and the formation of small vacuoles on the cell surface without obvious pyknosis of the nucleus; rupture or dissolution was found in the nucleus when it fell off into the intercellular space. In the PU-treated group, these changes were reversed. Furthermore, we detected pyroptosis-related proteins. A Western blot demonstrated that the expression of NLRP3, IL-1β, GSDMD, and caspase-1 in the kidneys of diabetic mice was markedly higher than in the control group (Figure 3D). These changes were reversed by treatment with PU. 

### 3.4. PU Attenuated the Expression of NOX4 and TXNIP

**Figure 4:**
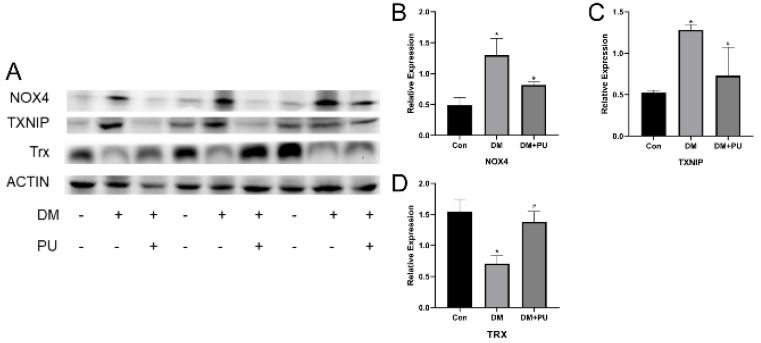
Effects of PU on the high glucose-induced inflammasome signaling pathway. (**A**) The results of NOX4, TXNIP, and Trx by Western blot. (**B**) Protein expression levels of NOX4 by WB. (**C**) Protein expression levels of TXNIP by WB. (**D**) Protein expression levels of Trx by WB. The relative expression is expressed by the ratio of the target protein to β-actin. * *p* < 0.05 vs. Con group; # *p* < 0.05 vs. DM group.

To explore how the inflammasome is activated, we examined the alterations in the levels of NOX4, TXNIP, and NLRP3 in the renal tissue of diabetic mice, measuring by Western blot (Figure 4). As an upstream protein that mediates the activation of the NLRP3 inflammasome, the expression of TXNIP was inhibited in the PU treatment group compared with the model group, which was consistent with the inhibition of pyroptosis and inflammation verified in the previous experiment. Trx could combine with TXNIP to make it inactive, and the rise in its expression in the PU treatment group also verified the above result.

## 4. Discussion

As one of the major microvascular complications of type 2 diabetes mellitus, diabetic nephropathy is a serious hazard [24]. Based on the mechanism of metabolic disorders and hemodynamics, the inflammatory mechanism is the critical factor for the occurrence and development of DN [3]. We used a chemical drug induction method: mice (C57/BL6) were fed a high-fat diet and injected with STZ intraperitoneally. This method simulates the formation of human type 2 diabetes. The low-dose injection of STZ also destroys some functions of islet β cells, reduces insulin secretion, and forms non-insulin-dependent (type 2) diabetes. We further confirmed that after several weeks, diabetic mice showed DN-significant pathological changes such as glomerulosclerosis, renal interstitial fibrosis, and deterioration of the renal function indicators, while the mice in the PU treatment group showed a marked reduction in renal damage.

The model of type 2-diabetic mice induced by STZ and a high-fat diet was used to evaluate and elucidate the protective effect and mechanism of PU on diabetic nephropathy. Based on this model, we explored the potential effect of PU on diabetic nephropathy and its underlying mechanism, proving that PU provides potential renal protection, which is characterized by inhibition of the urine albumin, serum creatinine, and BUN levels. Also, PU reduces glomerular hypertrophy, glomerular basement membrane expansion, and mesangial hyperplasia. These results indicate that in diabetic mice, the administration of PU can improve diabetic nephropathy.

Extensive studies have shown that the NLRP3 inflammasome detects endogenous danger signals, leading to the activation of caspase-1 and IL-1β; as a result, these events stimulate the inflammatory cascade, which is critical for DN [25]. Therefore, we investigated the role of the NLRP3 inflammasome in the pyroptosis pathway and its significance for DN in this study. In the hyperglycemic state, the body can generate a variety of risk-related molecules, such as fatty acids and reactive oxygen species, which can be recognized by the relevant PRRs, causing pyroptosis to start [26]. 

Although the health-beneficial capabilities of PU have been studied, its possible underlying mechanism is still full of uncertainty. The general trend is to explain the pharmacological effect of polyphenols by their antioxidant activity, although the concentration of polyphenols in the blood can hardly reach the level required for this effect (10–100 μM). Previous studies have shown that biological effects such as the anti-inflammatory effect of polyphenols are mediated by their interaction with specific plasma proteins or intracellular elements [27]. ECM observation of kidney tissue in diabetic mice in our research also confirmed this view. The NLRP3 inflammasome is able to detect endogenous danger signals, thus activating caspase-1 and IL-1β [28], closely related to the inflammatory cascade reaction in the process of DN. In our study, Western blotting and immunofluorescence results showed that the administration of PU could reduce the expression of pyroptosis-related proteins such as caspase-1, NLRP3, and GSDMD in renal tissue, and inhibit the expression of the downstream inflammatory molecule IL-1β, suggesting that PU could alleviate the inflammatory response by inhibiting pyroptosis [29]. 

With the development of diabetic nephropathy research, the significance of the Trx-TXNIP signaling system is increasingly recognized [30]. Recent studies have shown a complex thiol-dependent interaction between TXNIP and the inflammation-related pathway of progressive diabetic nephropathy [31], the interaction of NLRP3 and TXNIP being a significant signal of the formation of NOX4-derived NLRP3 inflammation in hyperhomocysteinemia-induced glomerular damage [32]. To verify whether the TXNIP-NLRP3 axis plays an important role in the occurrence of DN, we investigated the function of mitochondria and the expression of NLRP3, TXNIP, and IL-1 β. The results showed that consistent with pathological examination results and biochemical indicators, damage to mitochondria in the kidneys of diabetic mice was aggravated, and the expression level of these proteins was also up-regulated. Our findings confirm the previous view that mitochondrial dysfunction induced by hyperglycemia plays an essential part in the occurrence of DN, and are also consistent with the following hypothesis: the rise in the ROS level caused by mitochondrial damage plays a vital role in the pathogenesis of DN through the TXNIP-NLRP3 axis, and is likely to be related to pyroptosis [33].

As an important energy-supplying organelle, there are several oxidative pathways that exist in the mitochondria [34]. NADPH oxidases exist in many kinds of somatic cells including phagocytes. NOX4 is the most widely distributed of these oxidases and is the main source of ROS localized to mitochondria [35]. Moreover, there are considerable studies that support the position that an increase in ROS, in particular NADPH oxidase-derived ROS, is critical for the activation of the NLRP3 inflammasome. In addition, the inhibition of NOX4 effectively weakens ROS production and NLRP3, TXNIP, IL-1β, and caspase-1 protein expression in high glucose-treated mice, suggesting that NOX-induced ROS production could function through the TXNIP/NLRP3 axis [36,37]. Therefore, in this study, we mainly focused on clarifying that NOX4 activates the NLRP3 inflammasome and leads to pyroptosis, which is related to kidney damage in diabetic mice [38]. We consider that the inhibition of NOX expression and mtROS production in diabetic mice could result in sufficiently inhibiting NRLP3 inflammasome activation. Excessive mtROS production caused by NOX4 dissociated TXNIP from its binding protein Trx, which then bound to NLRP3 and promoted the activation of the NLRP3 inflammasome [39]. We used Western blotting to assess the expression of TXNIP and NOX4 and changes in TXNIP/NLRP3; the results show that treatment with PU reduces the expression of NOX4, and the expression of TXNIP and NLRP3 also decreased, which is in line with our expectation about the relationship between NOX4 and the TXNIP/NLRP3 pathway and the regulatory role of NOX4 in diabetic nephropathy. Under the regulation of NOX4, TXNIP may inhibit the activation of NLRP3 inflammatory bodies by dissociating from Trx and binding with NLRP3, and PU may play a meaningful role in protection against diabetic nephropathy. However, this hypothesis still needs to be verified by subsequent experiments.

## 5. Conclusions

In summary, we provide evidence that PU protects against DN. Inhibition of the inflammatory response caused by pyroptosis plays a vital part in the protective effects of PU. This process is driven by a novel mechanism, and the key to this mechanism is to reduce pyroptosis by inhibiting the TXNIP/NLRP3 axis. This study shows that the TXNIP/NLRP3 axis is an important pathway that regulates DN induced by pyroptosis, and that PU is a possible choice in the treatment of DN and other related diseases.

## Figures and Tables

**Table 1:** Glomerular tuft area of mice ($\bar{x}$ ± s, *n* = 8).

Group	Control	DM	DM+PU
Area (× 100 μm^2^)	20.95 ± 4.22	49.62 ± 4.74 *	29.10 ± 6.69 #

PU means punicalagin; * *p* < 0.05 vs. Con group; # *p* < 0.05 vs. diabetes mellitus (DM) group.

## References

[1] Pugliese G. (2014). Updating the natural history of diabetic nephropathy. Acta Diabetol..

[2] Umanath K., Lewis J.B. (2018). Update on diabetic nephropathy: Core curriculum 2018. Am. J. Kidney Dis..

[3] Moreno J.A., Gomez-Guerrero C., Mas S., Sanz A.B., Lorenzo O., Ruiz-Ortega M., Opazo L., Mezzano S., Egido J. (2018). Targeting inflammation in diabetic nephropathy: A tale of hope. Expert Opin. Investig. Drugs.

[4] Hu Z.B., Ma K.L., Zhang Y., Wang G.H., Liu L., Lu J., Chen P.P., Lu C.C., Liu B.C. (2018). Inflammation-activated CXCL16 pathway contributes to tubulointerstitial injury in mouse diabetic nephropathy. Acta Pharmacol. Sin..

[5] Shi J., Gao W., Shao F. (2017). Pyroptosis: Gasdermin-Mediated Programmed Necrotic Cell Death. Trends Biochem. Sci..

[6] Man S.M., Karki R., Kanneganti T.D. (2017). Molecular mechanisms and functions of pyroptosis, inflammatory caspases and inflammasomes in infectious diseases. Immunol. Rev..

[7] Hughes M.M., O’Neill L. (2018). Metabolic regulation of NLRP3. Immunol. Rev..

[8] Hutton H.L., Ooi J.D., Holdsworth S.R., Kitching A.R. (2016). The NLRP3 inflammasome in kidney disease and autoimmunity. Nephrology (Carlton).

[9] Qiu Y.Y., Tang L.Q. (2016). Roles of the NLRP3 inflammasome in the pathogenesis of diabetic nephropathy. Pharmacol. Res..

[10] Yu Z.W., Zhang J., Li X., Wang Y., Fu Y.H., Gao X.Y. (2020). A new research hot spot: The role of NLRP3 inflammasome activation, a key step in pyroptosis, in diabetes and diabetic complications. Life Sci..

[11] Lu J., Holmgren A. (2014). The thioredoxin antioxidant system. Free Radic. Biol. Med..

[12] Alhawiti N.M., Al M.S., Aziz M.A., Malik S.S., Mohammad S. (2017). TXNIP in metabolic regulation: Physiological role and therapeutic outlook. Curr. Drug Targets.

[13] Wen Y., Liu Y.R., Tang T.T., Pan M.M., Xu S.C., Ma K.L., Lv L.L., Liu H., Liu B.C. (2018). mROS-TXNIP axis activates NLRP3 inflammasome to mediate renal injury during ischemic AKI. Int. J. Biochem. Cell Biol..

[14] Kumar A., Mittal R. (2018). Mapping Txnip: Key connexions in progression of diabetic nephropathy. Pharmacol. Rep..

[15] Grosso G. (2018). Effects of Polyphenol—Rich foods on human health. Nutrients.

[16] Murakami A. (2018). Non-specific protein modifications may be novel mechanism underlying bioactive phytochemicals. J. Clin. Biochem. Nutr..

[17] Qiao S., Liu R., Lv C., Miao Y., Yue M., Tao Y., Wei Z., Xia Y., Dai Y. (2019). Bergenin impedes the generation of extracellular matrix in glomerular mesangial cells and ameliorates diabetic nephropathy in mice by inhibiting oxidative stress via the mTOR/beta-TrcP/Nrf2 pathway. Free Radic. Biol. Med..

[18] Karim N., Rahman A., Chanudom L., Thongsom M., Tangpong J. (2019). Mangosteen vinegar rind from Garcinia mangostana prevents high-fat diet and streptozotocin-induced type II diabetes nephropathy and apoptosis. J. Food Sci..

[19] Saltiel A.R., Olefsky J.M. (2017). Inflammatory mechanisms linking obesity and metabolic disease. J. Clin. Investig..

[20] Manna K., Mishra S., Saha M., Mahapatra S., Saha C., Yenge G., Gaikwad N., Pal R., Oulkar D., Banerjee K. (2019). Amelioration of diabetic nephropathy using pomegranate peel extract-stabilized gold nanoparticles: Assessment of NF-kappaB and Nrf2 signaling system. Int. J. Nanomed..

[21] Cao Y., Chen J., Ren G., Zhang Y., Tan X., Yang L. (2019). Punicalagin Prevents Inflammation in LPS-Induced RAW264.7 Macrophages by Inhibiting FoxO3a/Autophagy Signaling Pathway. Nutrients.

[22] Xu X., Li H., Hou X., Li D., He S., Wan C., Yin P., Liu M., Liu F., Xu J. (2015). Punicalagin Induces Nrf2/HO-1 expression via upregulation of PI3K/AKT pathway and inhibits LPS—induced oxidative stress in RAW264.7 macrophages. Mediat. Inflamm..

[23] Zhang Y., Cao Y., Chen J., Qin H., Yang L. (2019). A new possible mechanism by which punicalagin protects against liver injury induced by type 2 diabetes mellitus: Upregulation of autophagy via the Akt/FoxO3a Signaling Pathway. J. Agric. Food Chem..

[24] Papadopoulou-Marketou N., Chrousos G.P., Kanaka-Gantenbein C. (2017). Diabetic nephropathy in type 1 diabetes: A review of early natural history, pathogenesis, and diagnosis. Diabetes Metab. Res. Rev..

[25] Shahzad K., Bock F., Dong W., Wang H., Kopf S., Kohli S., Al-Dabet M.M., Ranjan S., Wolter J., Wacker C. (2015). Nlrp3-inflammasome activation in non-myeloid-derived cells aggravates diabetic nephropathy. Kidney Int..

[26] Haque R., Iuvone P.M., He L., Hur E.H., Chung C.K., Park D., Farrell A.N., Ngo A., Gokhale S., Aseem M. (2017). Prorenin receptor (PRR)-mediated NADPH oxidase (Nox) signaling regulates VEGF synthesis under hyperglycemic condition in ARPE-19 cells. J. Recept. Signal. Transduct. Res..

[27] Williamson G., Clifford M.N. (2017). Role of the small intestine, colon and microbiota in determining the metabolic fate of polyphenols. Biochem. Pharmacol..

[28] Wang S., Li Y., Fan J., Zhang X., Luan J., Bian Q., Ding T., Wang Y., Wang Z., Song P. (2017). Interleukin-22 ameliorated renal injury and fibrosis in diabetic nephropathy through inhibition of NLRP3 inflammasome activation. Cell. Death. Dis..

[29] Lyu A., Chen J.J., Wang H.C., Yu X.H., Zhang Z.C., Gong P., Jiang L.S., Liu F.H. (2017). Punicalagin protects bovine endometrial epithelial cells against lipopolysaccharide—Induced inflammatory injury. J. Zhejiang Univ. Sci. B.

[30] Ji L., Wang Q., Huang F., An T., Guo F., Zhao Y., Liu Y., He Y., Song Y., Qin G. (2019). FOXO1 overexpression attenuates tubulointerstitial fibrosis and apoptosis in diabetic kidneys by ameliorating oxidative injury via TXNIP-TRX. Oxid. Med. Cell. Longev..

[31] Xu W., Wang L., Li J., Cai Y., Xue Y. (2018). TXNIP mediated the oxidative stress response in glomerular mesangial cells partially through AMPK pathway. Biomed. Pharmacother..

[32] Abais J.M., Xia M., Li G., Chen Y., Conley S.M., Gehr T.W., Boini K.M., Li P.L. (2014). Nod-like receptor protein 3 (NLRP3) inflammasome activation and podocyte injury via thioredoxin-interacting protein (TXNIP) during hyperhomocysteinemia. J. Biol. Chem..

[33] Han Y., Xu X., Tang C., Gao P., Chen X., Xiong X., Yang M., Yang S., Zhu X., Yuan S. (2018). Reactive oxygen species promote tubular injury in diabetic nephropathy: The role of the mitochondrial ros-txnip-nlrp3 biological axis. Redox Biol..

[34] Reczek C.R., Chandel N.S. (2015). ROS-dependent signal transduction. Curr. Opin. Cell. Biol..

[35] Shanmugasundaram K., Nayak B.K., Friedrichs W.E., Kaushik D., Rodriguez R., Block K. (2017). NOX4 functions as a mitochondrial energetic sensor coupling cancer metabolic reprogramming to drug resistance. Nat. Commun..

[36] Gu C., Draga D., Zhou C., Su T., Zou C., Gu Q., Lahm T., Zheng Z., Qiu Q. (2019). miR-590-3p Inhibits Pyroptosis in Diabetic Retinopathy by Targeting NLRP1 and Inactivating the NOX4 Signaling Pathway. Investig. Ophthalmol. Vis. Sci..

[37] Wang W., Wu Q.H., Sui Y., Wang Y., Qiu X. (2017). Rutin protects endothelial dysfunction by disturbing Nox4 and ROS-sensitive NLRP3 inflammasome. Biomed. Pharmacother..

[38] Li X., Cai W., Lee K., Liu B., Deng Y., Chen Y., Zhang X., He J.C., Zhong Y. (2017). Puerarin attenuates diabetic kidney injury through the suppression of NOX4 expression in podocytes. Sci. Rep..

[39] Shah A., Xia L., Masson E.A., Gui C., Momen A., Shikatani E.A., Husain M., Quaggin S., John R., Fantus I.G. (2015). Thioredoxin—Interacting protein deficiency protects against diabetic nephropathy. J. Am. Soc. Nephrol..

